# Ovarian Cancer Stem Cells: A New Target for Cancer Therapy

**DOI:** 10.1155/2013/916819

**Published:** 2013-01-30

**Authors:** Qinglei Zhan, Chunmei Wang, Saiming Ngai

**Affiliations:** School of Life Sciences, The Chinese University of Hong Kong, New Territories, Hong Kong

## Abstract

Ovarian cancer is a highly lethal disease among all gynecologic malignancies and is the fifth leading cause of cancer-related death in women. Although the standard combination of surgery and chemotherapy was initially effective in patients with ovarian cancer, disease relapse commonly occurred due to the generation of chemoresistance. It has been reported that cancer stem cells (CSCs) are involved in drug resistance and cancer recurrence. Over the past decades, increasing studies have been done to identify CSCs from human ovarian cancer cells. The present paper will summarize different investigations on ovarian CSCs, including isolation, mechanisms of chemoresistance, and therapeutic approaches. Although there are still numerous challenges to translate basic research to clinical applications, understanding the molecular details of CSCs is essential for developing effective strategies to prevent ovarian cancer and its recurrence.

## 1. Introduction

Ovarian cancer, the fifth leading cause of cancer-related death in women, is a highly lethal disease among all gynecologic malignancies. It is estimated that 22,280 women are diagnosed with ovarian cancer and 15,500 women will die of this disease in 2012 in the United States. From 2005 to 2009, the median age at diagnosis for ovarian cancer in women was 63 years. Based on incidences from 2007 to 2009, one in seventy-two women will be diagnosed with ovarian cancer during their lifetime. The overall five-year relative survival rate was 43.7% from 2002 to 2008 [1]. 

Ovarian cancer is a heterogeneous disease composed of different types of tumors [2]. Based on different histological features, most tumors of the ovary contain three major types of cells: surface epithelial stromal cells, sex cord stromal cells (including granulose, theca, and hilus cells), and germ cells (oocytes) [3]. Epithelial ovarian carcinoma (EOC) is the major form of the disease and accounts for about 90% of ovarian tumors [4]. According to distinctive morphology and molecular genetic background, epithelial ovarian cancer can be further categorized into eight subtypes, including serous, mucinous, endometrioid, clear cell, transitional cell tumors (Brenner tumors), carcinosarcoma, mixed epithelial tumor, and undifferentiated carcinoma [5]. Various subtypes of epithelial ovarian cancers can be also simply divided into two groups named type I and type II by Kurman and Shih in 2010 [6]. Type I tumors are clinically indolent and genetically stable, including low-grade serous, low-grade endometrioid, clear cell, and mucinous and transitional (Breener) carcinomas. Type II tumors are more aggressive and genetically unstable, including high-grade serous, high-grade endometrioid, carcinosarcoma, mixed epithelial tumor, and undifferentiated carcinomas [7].

Over the past decades, the combination of surgery and platinum-based chemotherapy was the standard treatment for advanced ovarian cancer [8]. Although numerous molecular targeting agents have been developed due to deeper understanding of the disease progression, recurrence still commonly occurs in 70% of patients who underwent the first-line treatment within 18 months. The five-year survival rate of those patients with advanced ovarian cancer is only 30.6% [9, 10]. Thus it is crucial to develop effective strategies to attack cancer cells that become resistant to current chemotherapy. 

Recently, scientists have proposed that the existence of cancer stem cells was one of the reasons for disease relapse [11, 12]. Traditional chemotherapy can kill the majority of cancer cells, while failing to target cancer stem cells. Moreover, initial treatment increased the proportion of drug-resistant cancer stem cells, resulting in recurrence of disease [13]. In this paper, we will summarize the studies on ovarian CSCs, including the isolation, their roles in chemoresistance, and the therapeutic approaches.

## 2. Cancer Stem Cells of Ovaries

The terms cancer stem cells (CSCs) or cancer initiating cells (CICs) are a very small subgroup of tumor cells with the ability to self-renew, differentiate, and form secondary/tertiary tumors after serial xenotransplantation into immune-compromised animal models [14, 15]. Actually, the reason for 90% of tumors arising from ovary surface epithelium is that stem cells reside in the area. In early stage of ovarian cancer, the number of EOC stem cells can be used to predict progression of the disease [16]. 

Understanding the origin of cancer cells may have clinical significance. It has been reported that both luminal and basal epithelial cells are cells of origin for prostate cancer [17, 18]. In the case of CSCs, it originated not only from adult stem cells that underwent oncogenic transformation, but also from downstream progenitor or differentiated cells with acquired stem cell-like characteristics [19]. However, limited evidence suggested that adult stem cells were the originator of ovarian cancer. Tumors arising from CSCs usually contain a mixed population of cells due to asymmetric division of CSCs. Such cell division can produce one daughter cell that retains the feature of parent cell and another that continually divides to form the bulk of tumor [20]. 

In 1997, Bonnet and Dick first isolated the cancer stem cells in leukemic cells expressing stem cell marker CD34 [21]. Later, many other types of CSCs were also identified, including ovarian CSCs [22]. The first evidence of ovarian stem cells was the isolation of the ascites from a patient with ovarian cancer [23]. One of the properties for stem cells is to exclude harmful dyes, thus containing less cytoplasmic dyes compared to the rest of the cell population by fluorescence-activated cell sorting (FACS) analysis [24]. Ovarian cancer stem cells can be successfully isolated via distinctive efflux of the DNA binding dye Hoechst 33342. These ovarian CSCs are also called “side population” (SP) stem cells that have the capacity of self-renewal and differentiation in comparison with the non-SPs [25]. However, there is no universal single marker for ideally isolating the ovarian CSCs. In 2009, Gao and his colleagues have isolated SP cells from OVCAR-3, a human ovarian cancer cell line. However, these cell fractions only accounted for 0.9% of the total cell populations [26]. Another study successfully established stable SP cells and ALDH1A1 positive cell populations from the ovarian cancer cell line A2780. Those SP cells exhibited partial resistance to the chemotherapy drug platinum. Nevertheless, it should be noted that a cancer stem cell population may not be a group of cells with a single feature, but may contain overlapping cell fractions with mixed stem-like markers [27]. 

## 3. Chemoresistance of Ovarian CSCs

Although the standard combination of surgery and chemotherapy can effectively reduce tumor mass, most patients with residual ovarian CSCs eventually acquire chemoresistance. Hence, recurrent cancer is inevitable in the vast majority of cases [28, 29]. Such phenomenon attracts researchers' attention to decipher the molecular mechanisms involved in escaping the chemotherapy for cancer stem cells.

### 3.1. Glutathione (GSH) System

The GSH system can suppress oxidative stress and maintain cellular redox homeostasis [30]. The contribution of GSH and GSH-related enzymes to chemoresistance has been demonstrated in different types of tumor, including ovarian cancer and brain tumor [31, 32]. GSH is also involved in the detoxification of various xenobiotics [33]. Upon metabolism of chemotherapeutic agents, the enzymes of glutathione-S-transferase (GST) family could prompt the formation of GSH-drug conjugates. Many chemotherapeutic agents have been shown to conjugate with GSH, including chloroethylnitrosoureas (CENUs), platinum compounds, and other alkylating agents. The resulting GSH-drug conjugates are more water soluble and less active than the compounds themselves. They are thus exported from the cell via the transporter-mediated system [34]. These findings reasonably support the application of antioxidant inhibitors, in combination with standard chemotherapy in patients. 

### 3.2. Overexpression of Bmi-1

Bmi-1, a member of the polycomb group (PcG) family, participates in the self-renewal and maintenance of CSCs [35]. As an oncogene, Bmi-1 could enable cancer cells to escape apoptosis by modulating multiple growth signaling pathways [36]. Thus, its overexpression in cancer cells could be used as a survival marker. The role of Bmi-1 in chemoresistance has been addressed recently. For example, Bmi-1 could allow the resistance of glioma cells to chemotherapy drug such as doxorubicin and bis-chloroethylnitrosourea (BCNU) [37]. It can also prompt chemoresistance, invasion and tumorigenesis in pancreatic cancer cells [38]. For ovarian cancer cells, silencing of Bmi-1 gene could promote sensitivity to cisplatin and induction of apoptosis [39].

### 3.3. Loss and Localization of p53

The tumor suppressor gene p53 plays a critical role in cell proliferation and apoptosis by controlling several signaling pathways. Loss of p53 function could cause multidrug resistance in many types of tumors, including ovarian cancer [40]. In addition, the control of intracellular localization of p53 is also associated with the regulation of apoptosis and chemosensitivity in human ovarian cancer cells [41]. The p53-associated Parkin-like cytoplasmic (PARC) protein is critical for p53 subcellular localization and function. It has been demonstrated that a low level of PARC could increase p53 accumulation in nucleus, thus inducing apoptosis [42]. Downregulation of Ca^2+^-dependent PARC could enhance cisplatin-induced apoptosis in chemosensitive but not in chemoresistant human ovarian cancer cells [43]. The detailed molecular mechanism affecting PARC/p53 interaction between chemosensitive and chemoresistant cancer cells remains to be determined. However, it is vital to note that p53 is not an absolute indicator for the resulting response to chemotherapy because not all drugs induce cell death via p53 in cancer cells [44].

### 3.4. Drug Effluxion

The development of multidrug resistance is also associated with the failure of drug uptake. The export of drugs is mediated by transmembrane polysubstrate efflux pumps, which prevent drugs from entering their intracellular targets [45]. These drug transporters are composed of four domains, including two nucleotide-binding domains (NBD) and two transmembrane domains (TMD). The TMD recognizes and translocates substrates, while the NBD is required for conformational changes [46]. 

Mammalian P-glycoprotein is a transmembrane transporter related with resistance of hydrophobic anticancer drugs. It belongs to one of the ATP-binding cassette (ABC) transporter families [47]. For decades, other efflux transporters in the ABC transporter family have been also identified. For example, ABBC2 encoding for MRP2 (multidrug resistance protein) was involved in effluxion of cisplatin-derived compound in ovarian cancers [48]. ABCG2 (breast cancer resistance protein or BCRP) permitted effluxion of cellular DNA-binding dye Hoechst. Thus, Hoechst can be used to isolate stem-like cells in a variety of tissues, including bone marrow, skeletal muscle, mammary epithelium [49, 50], and ovarian carcinomas [51]. Moreover, ABCG2/BCRP was considered as a drug-resistant marker, which involved in transport of substances and cellular products by using ATP as energy source [52]. In addition to the ABC family, some other transporters have been described such as copper transporter proteins (CTR), organic cation transporters (OCTs), copper-transporting ATPases, and multidrug and toxin extrusion (MATE) [53].

Wender and his colleagues recently conjugated a known drug (Taxol) to oligoarginine, which is a guanidinium-rich molecular transporter responsible for delivery of attached molecule into cells. Such Taxol-oligoarginine conjugates may overcome drug efflux-based resistance through prolonging the half-life of the drug and increasing the drug stability in human ovarian carcinoma cells [54].

### 3.5. Quiescence of Ovarian CSCs

Mammalian adult stem cells are known to maintain in a quiescent, nondividing, or G0 state [55]. CSCs also demonstrated the similar property. This is also one of the reasons for their resistance to chemotherapy since most anticancer drugs preferentially target dividing cancer cells. Thus, intensive understanding of quiescent mechanism of CSCs is important to improve clinical outcome for cancer patients. 

Recent studies have suggested that several genes played key roles in maintaining quiescence of normal stem cells and CSCs. For example, p53 expression was increased and could promote quiescence in hematopoietic stem cells (HSCs) [56]. Necdin, a growth-suppressing protein, as well as a p53 target gene, has been recently identified to improve hematopoietic stem cells quiescence [57]. Nonetheless, the loss of zinc-finger repressor Gfi-1 (growth factor independent 1) enabled HSCs high proliferation [58]. Cited2, a transcriptional modulator, could maintain HSCs quiescence via both HIF-1 (a negative regulator) dependent and independent pathways. Deletion of Cited2 could improve HSCs apoptosis and loss of quiescence. Moreover, its deletion could increase cycling in conditional knockout mice [59]. In addition, the reduced miRNAs (miR-31 and let-7) were demonstrated to keep the balance between lung cancer stem-like side population (SP) cells and nonside population (non-SP) cells. Inhibition of let-7 could prompt growth of both SP and non-SP cells by accelerating G1 to S phase transition, while repression of miR-31 could cause cell cycle arrest in G0/G1 phase in both of SP and non-SP cells [60].

## 4. Therapeutic Approaches of Ovarian CSCs

The elimination of ovarian CSCs has been challenging in part due to heterogeneity. Thus the efficacy of any single drug was limited for cancer patients. Combined treatments that target CSCs will be a new direction in the future. Nevertheless, drug treatment for CSCs may increase the risk of toxicity since CSCs share common features with normal stem cells. The current therapeutic strategies in ovarian CSCs are discussed below.

### 4.1. Cell Surface and Nonsurface Markers

Cell surface markers (i.e., CD molecules, short for cluster of differentiation) have been widely used to isolate putative CSCs through flow cytometry. Most types of CSCs share the identical biomarkers, including ovarian cancer stem cells. To activate the immune system to clear cancer cells in patient body, antibody-based therapy for cancer has been developed for decades. Moreover, the strategy of antibody-drug conjugates has achieved considerable success in recent years [61]. Indeed, development of specific therapies that target biomarkers of ovarian CSCs could improve clinical outcome and patient's survival [62]. 

#### 4.1.1. CD133

CD133, a transmembrane glycoprotein, is one of the most widely described ovarian CSCs markers [63]. Its expression level is higher in advanced serous ovarian cancer than that in normal ovaries and benign tumors [62]. Tumor cells carrying CD133 marker (often abbreviated as CD133^+^) displayed greater resistance to chemotherapy [63]. In addition, CD133^+^ ovarian CSCs have hyperactivity in migration and invasion due to the activation of chemokine (c-c motif) ligand 5 (CCL5) [64]. In 2009, Baba and his colleagues found that methylation in promoter region could regulate the expression of CD133 in ovarian cancers, implying that epigenetic modification might be involved in the induction of stemness of tumor [65]. In addition, the combination of a murine derived anti-human CD133 antibody and a cytotoxic drug (monomethyl auristatin F, MMAF) significantly inhibited the cell growth in hepatocellular and gastric cancers [66].

#### 4.1.2. CD44

CD44, another CSC surface transmembrane glycoprotein, is a receptor for hyaluronic acid (HA) involved in cell-cell and cell-matrix interactions. It will ultimately affect cellular growth, differentiation, and motility [67, 68]. CD44 is highly expressed in many types of cancer, including ovarian CSCs. The CD44^+^/CD24^−^ ovarian cancer cells were correlated with invasion and chemoresistance [69]. Several antibodies against isoforms of CD44 have been developed, and some of them have entered into clinical trials for the patients with head and neck squamous cancers [70]. VFF18 and BIWA-1 were two murine IgG1 monoclonal antibodies that recognized human CD44 variant exon 6 (CD44v6). They were evaluated for their targeting potential in squamous cell carcinoma (SCC) and head and neck SCC (HNSCC), respectively. To avoid human anti-mouse antibody response in patients, humanized forms of such antibodies were developed, such as BIWA-2, BIWA-4 and BIWA-8 [71]. The phase I clinical trial of BIWA-4 (bivatuzumab) has been carried out to evaluate its safety, tumor-targeting potential, pharmacokinetics, and immunogenicity in patients with HNSCC [72]. However, these clinical outcomes still need further confirmation. Except for antibody-based therapy, scientists also proposed other approaches in recent years. Casagrande and his colleagues reported that a toxin called clostridium perfringens enterotoxin (CPE) could eradicate chemoresistant CD44^+^ ovarian CSCs in mouse xenograft model [73]. In addition, the conjugate of hyaluronic acid to paclitaxel has been also tried for the treatment of ovarian cancer [74]. 

#### 4.1.3. CD24

CD24 is a glycosylphosphatidylinositol-linked cell surface protein expressed in various solid tumors [75]. Gao et al. have successfully isolated CD24^+^ CSCs from ovarian tumor specimens and identified CD24 as a putative CSC marker in ovarian cancer [76]. Expression of CD24 affected metastasis and represented poor prognosis in ovarian cancer [77]. A study demonstrated that CD24 could localize in the cytoplasm of ovarian serous tumors, while normal epithelium and serous cystadenomas expressed CD24 marker in the apical membrane. Thus, the cytoplasmic expression of CD24 could be used as a specific marker to predict the survival rates and recurrence of cancer [78]. The depletion and over-expression of CD24 could regulate the phosphorylation of STAT3 and FAK by affecting Src (nonreceptor tyrosine kinases) activity. SWA11, an antibody against CD24 reduced tumor size in xenograft mice transplanted by lung cancer cells A549 and pancreatic cancer cells BxPC3 [79]. In 2009, Su and his colleagues successfully applied short hairpin RNA (shRNA) to reduce CD24 expression. The knockdown of CD24 decreased cell viability by activation of apoptosis in ovarian cell line SKOV3 in vitro and also suppressed tumor growth in nude mice bearing ovarian cancer in vivo [80]. Therefore, CD24 inhibition may be considered as an effective approach for cancer therapy.

#### 4.1.4. CD117

CD117, known as c-kit, is a type III receptor tyrosine kinase involved in cell signal transduction. It involved in various cellular processes, including apoptosis, cell differentiation, proliferation, and cell adhesion [81]. High expression level of CD117 was observed in ovarian cancers [82]. Luo and his colleagues further demonstrated that as few as 10^3^ CD117^+^ ovarian cancer cells had the ability to self-renew, differentiate, and regenerate tumor in xenograft model [83]. It has been also suggested that CD117 in ovarian carcinoma was associated with poor response to chemotherapy [84]. The activation of Wnt/*β*-catenin-ATP-binding cassette G2 pathway was required for cisplatin/paclitaxel-based chemoresistance caused by CD117 in ovarian CSCs [85]. A potent CD117 specific inhibitor (Imatinib Mesylate) has been used in the clinical trials for the treatment of many types of cancer, including persistent epithelial ovarian cancer [86]. Patel and his colleagues demonstrated that Imatinib Mesylate involved in complex cellular processes, including metabolic pathways, cell cycle, cell proliferation, apoptosis, and signal transduction through mass spectrometry-based proteomics method in human ovarian cancer cell line A2780 [87]. 

#### 4.1.5. EpCAM

The epithelial cell adhesion molecule EpCAM is a glycosylated membrane protein. It is highly expressed in different tumor types, including colon, lung, pancreas, breast, head and neck, and ovary [88]. EpCAM was found to be hyperglycosylated and frequently associated with cytoplasmic staining in carcinoma tissues [89, 90]. EpCAM is comprised of an extracellular domain (EpEX), a single transmembrane domain and a short 26-amino acid intracellular domain (EpICD). Among them, EpEX is required for cell-cell adhesion [91]. Downregulation of EpCAM could cause loss of cell-cell adhesion and promote epithelial mesenchymal transition (EMT). Metastasis thus occurred in carcinomas [92]. EpCAM positive cells also have tumor-initiating potential, making it a potential target for cancer therapy. Catumaxomab, a monoclonal antibody against EpCAM is a trifunctional antibody, which can bind three different cell types, including tumor cells, T cells, and accessory cells (dendritic cell, macrophages, and natural killer cells) [93]. It is now used in phase III clinical trials in patients with malignant ascites [94]. The investigation of its efficacy and safety was also entered in phase II clinical trials on advanced ovarian cancer patients who had experienced complete chemotherapy. Based on both preclinical and clinical outcomes, EpCAM may be served as a possible therapeutic target against epithelial ovarian cancer.

#### 4.1.6. Aldehyde Dehydrogenase (ALDH) Isozymes

ALDH proteins are a superfamily containing 19 enzymes that protect cells from carcinogenic aldehydes [95]. ALDH1A1 was identified as a putative cancer stem cell marker, and it was associated with chemoresistance in the ovarian CSC [96]. Besides ALDH1A1, other ALDH isozymes such as ALDH1A3, ALDH3A2, and ALDH7A1 also had high expression level in ovarian tumors when compared to normal ovarian tissues [97]. The dual positivity of ALDH and CD133 ovarian cells had higher ability to regenerate tumor in mice than single ALDH^+^ or CD133^+^ ovarian cancer cells [98]. These findings suggest that ALDH can be used as a reliable marker to study ovarian cancer stem cells.

Recently, clinical trials have been initiated using disulfiram (an ALDH inhibitor). The combination of disulfiram with a drug named gemcitabine had a synergistic effect on cytotoxicity in glioblastoma multiforme cells [99]. A novel class of ALDH inhibitor (Aldi) discovered recently could endow lung cancer cell line A549 with higher sensitivity to mafosfamide [100].

Other two stem cell markers, Lin28 and Oct4, are also served as new molecular targets due to their roles in the maintenance of pluripotency in ovarian cancer [101]. In addition, high expression of the Müllerian inhibiting substance (MIS) type II receptor has been reported in ovarian cancer cell lines [102]. MIS could significantly inhibit the cell population with stem-like characteristics in ovarian cancer cell lines [103].

### 4.2. Differentiation of Ovarian CSCs

Current methods to eliminate CSCs cannot be successfully applied in all clinical situations. One way to eradicate CSCs is to induce their differentiation, resulting in loss of their stemness property [104]. Thus, the understanding of regulation of differentiation processes is necessary for designing new agents to eliminate CSCs. In 2012, Yin and his colleagues observed that TWIST-1 (a basic helix-loop-helix transcription factor) played a key role in triggering differentiation of epithelial ovarian cancer (EOC) [105]. Jain et al. recently reported that p53 capable for regulating molecular networks can activate two miRNAs (miR-34a and miR-145). These miRNAs were then shown to prompt differentiation of human embryonic stem cells [106]. Indeed, emerging evidence indicated that miRNAs were involved in self-renewal and differentiation of normal and cancer stem cells. It is suggested that such miRNAs should be a new therapeutic target for cancer treatment [107].

Retinoic acid (a vitamin A metabolite) and its analogs are the most common differentiation agents. They are also the only agents used in clinical trials [108]. The all-trans-retinoic acid (ATRA) can inhibit the proliferation and induce the differentiation via inhibition of Wnt/*β*-catenin pathway in head and neck squamous carcinoma CSC [109]. The clinical study of ATRA has shown an increased survival rate of patients with acute promyelocytic leukemia. However, successful cases are limited in solid tumors [110]. Recently, Whitworth and his colleagues effectively reduced the growth of ovarian CSC via a drug (Carboplatin) combined with three novel retinoid compounds [111]. In addition, specific unsaturated fatty acids (palmitoleic, oleic, and linoleic acids) can trigger adipocyte-like differentiation in many types of cancer cells, including ovarian cancer cell line SKOV3 [112]. However, more detailed regulation of differentiation remains to be determined.

### 4.3. Niches of CSCs

Niches are microenvironments where CSCs reside, containing cell-cell, cell-extracellular matrix, and soluble factors that support the growth, progression, and metastasis of CSCs [113]. Bone-marrow-derived mesenchymal stem cells (MSCs) are known to form fibroblast and myofibroblast populations in the tumor-associated stroma. Recently, evidence has been demonstrated that MSC and derived cell types could secrete prostaglandin E2 and release various cytokines, which is vital for the formation and progression of a tumor [114]. Furthermore, MSC affected metastatic ability and chemoresistance in two ovarian cancer cell lines: OVCAR3 and SKOV3 [115]. Katz et al. reported that tumorigenic ability of ovarian tumor cells was dependent on niches derived from human embryonic stem cells [116]. The hypoxic niches were beneficial for acquirement of stem-like properties of ovarian cancer cells [117]. 

These findings highlight the vital role of CSCs niches, which represent a promising therapeutic target for eradicating CSCs in the future. Indeed, disrupting components in the niches may yield better outcomes without noncytotoxic effect, when compared with that of removing the CSCs [118].

### 4.4. MicroRNAs (miRNAs)

MiRNAs are a group of small noncoding RNAs with 20–28 nucleotides in length. They could regulate gene expression at posttranscriptional level. Thus, miRNAs are involved in diverse biological processes, such as development and tumorigenesis [119]. The expression profile of miRNAs was different between normal stem cells and CSCs [120, 121]. MiR-214 was highly expressed in ovarian CSCs and endowed the property of self-renewal and chemoresistance in ovarian CSCs via repressing p53-Nanog pathway [122]. MiR-199a significantly rescued the sensitivity of ovarian CSCs to some chemotherapy agents, including cisplatin, paclitaxel, and Adriamycin. Moreover, miR-199a prevented tumorigenesis in xenograft model via downregulating expression of CSCs marker CD44 [123]. In addition, the expression of miR-200a could reduce migrating ability of CD133^+^ ovarian CSCs. This was because miR-200a inhibited E-cadherin and ZEB2, two genes critical for migration process [124]. However, some miRNAs own oncogenic property, such as miR-125, miR-9, miR-30, miR-21, and miR-215 [125, 126]. In conclusion, miRNAs have become a potential target for ovarian cancer treatment.

## 5. Conclusion

Understanding the roles of CSCs in cancer therapy may markedly improve the survival rate of ovarian cancer patients. However, it is impossible to cure patients with advanced ovarian cancer in all cases. One possible reason is the heterogeneity of ovarian CSCs, which leads to different sensitivities to the therapy used for one subset of CSCs. Thus combinative therapy will be the major direction for ovarian cancer treatment in the future. In addition, personalized medicine dependent on different genomic background of individuals will become a more effective therapeutic method. Current technological advances, such as next-generation DNA sequencing and mass spectrometry- (MS-) based proteomics, would facilitate implementation of personalized medicine. The establishment of comprehensive gene/protein network from cancer patients could provide more accurate platform for clinical prognosis [127, 128]. In 2012, Vathipadiekal and his colleagues have reported the gene expression profile in ovarian cancer stem cells by affymetrix microarray and identified the activation of Notch signaling pathway, as well as several other genes unique to ovarian CSCs [129]. 

In brief, we have highlighted the recent advances on ovarian CSCs, including isolation, mechanisms of chemoresistance, and therapeutic strategies. It is easy to imagine that understanding of the CSCs will be helpful to guide medical decision. Basic research is also fundamental to develop new agents for patients. It is our hope that therapies that target ovarian CSCs will result in better clinical outcomes.

## Abbreviations

CSCs: Cancer stem cells
EOC: Epithelial ovarian carcinoma
SP: Side population
GSH: Glutathione
PARC: p53-associated Parkin-like cytoplasmic
ABC: ATP-binding cassette
MRP: Multidrug resistance protein
BCRP: Breast cancer resistance protein
HSCs: Hematopoietic stem cells
HA: Hyaluronic acid
SCC: Squamous cell carcinoma
HNSCC: Head and neck SCC
CPE: Clostridium perfringens enterotoxin
EpCAM: Epithelial cell adhesion molecule
EMT: Epithelial mesenchymal transition
ALDH: Aldehyde dehydrogenase
MSC: Mesenchymal stem cells.

## References

[1] Howlader N, Noone AM, Krapcho M (1975/2009). *SEER Cancer Statistics Review*.

[2] Karst AM, Drapkin R (2010). Ovarian cancer pathogenesis: a model in evolution. *Journal of Oncology*.

[3] Chen VW, Ruiz B, Killeen JL (2003). Pathology and classification of ovarian tumors. *Cancer*.

[4] Bast RC, Hennessy B, Mills GB (2009). The biology of ovarian cancer: new opportunities for translation. *Nature Reviews Cancer*.

[5] Kaku T, Ogawa S, Kawano Y (2003). Histological classification of ovarian cancer. *Medical Electron Microscopy*.

[6] Kurman RJ, Shih IM (2010). The origin and pathogenesis of epithelial ovarian cancer: a proposed unifying theory. *American Journal of Surgical Pathology*.

[7] Kurman RJ, Visvanathan K, Roden R, Wu TC, Shih IM (2008). Early detection and treatment of ovarian cancer: shifting from early stage to minimal volume of disease based on a new model of carcinogenesis. *American Journal of Obstetrics and Gynecology*.

[8] Kim A, Ueda Y, Naka T (2012). Therapeutic strategies in epithelial ovarian cancer. *Journal of Experimental & Clinical Cancer Research*.

[9] Ferlay J, Parkin DM, Steliarova-Foucher E (2010). Estimates of cancer incidence and mortality in Europe in 2008. *European Journal of Cancer*.

[10] Leitao MM, Chi DS (2009). Surgical management of recurrent ovarian cancer. *Seminars in Oncology*.

[11] Kim CF, Dirks PB (2008). Cancer and stem cell biology: how tightly intertwined?. *Cell Stem Cell*.

[12] Ahmed N, Abubaker K, Findlay J (2013). Cancerous ovarian stem cells: obscure targets for therapy but relevant to chemoresistance. *Journal of Cellular Biochemistry*.

[13] Valent P, Bonnet D, De Maria R (2012). Cancer stem cell definitions and terminology: the devil is in the details. *Nature Reviews Cancer*.

[14] Gupta PB, Chaffer CL, Weinberg RA (2009). Cancer stem cells: mirage or reality?. *Nature Medicine*.

[15] Kitamura H, Okudela K, Yazawa T, Sato H, Shimoyamada H (2009). Cancer stem cell: implications in cancer biology and therapy with special reference to lung cancer. *Lung Cancer*.

[16] Steffensen KD, Alvero AB, Yang Y (2011). Prevalence of epithelial ovarian cancer stem cells correlates with recurrence in early-stage ovarian cancer. *Journal of Oncology*.

[17] Shibata M, Shen MM (2012). The roots of cancer: stem cells and the basis for tumor heterogeneity. *BioEssays*.

[18] Wang X, Julio MKD, Economides KD (2009). A luminal epithelial stem cell that is a cell of origin for prostate cancer. *Nature*.

[19] Barker N, Clevers H (2007). Tracking down the stem cells of the intestine: strategies to identify adult stem cells. *Gastroenterology*.

[20] Guddati AK (2012). Ovarian cancer stem cells: elusive targets for chemotherapy. *Medical Oncology*.

[21] Bonnet D, Dick JE (1997). Human acute myeloid leukemia is organized as a hierarchy that originates from a primitive hematopoietic cell. *Nature Medicine*.

[22] Zhang S, Balch C, Chan MW (2008). Identification and characterization of ovarian cancer-initiating cells from primary human tumors. *Cancer Research*.

[23] Bapat SA, Mali AM, Koppikar CB, Kurrey NK (2005). Stem and progenitor-like cells contribute to the aggressive behavior of human epithelial ovarian cancer. *Cancer Research*.

[24] Lin KK, Goodell MA (2006). Purification of hematopoietic stem cells using the side population. *Methods in Enzymology*.

[25] Szotek PP, Pieretti-Vanmarcke R, Masiakos PT (2006). Ovarian cancer side population defines cells with stem cell-like characteristics and Mullerian inhibiting substance responsiveness. *Proceedings of the National Academy of Sciences of the United States of America*.

[26] Gao Q, Geng L, Kvalheim G, Gaudernack G, Suo Z (2009). Identification of cancer stem-like side population cells in ovarian cancer cell line OVCAR-3. *Ultrastructural Pathology*.

[27] Zeimet AG, Reimer D, Sopper S (2012). Ovarian cancer stem cells. *Neoplasma*.

[28] Ahmed N, Abubaker K, Findlay J, Quinn M (2010). Epithelial mesenchymal transition and cancer stem cell-like phenotypes facilitate chemoresistance in recurrent ovarian cancer. *Current Cancer Drug Targets*.

[29] Hu L, McArthur C, Jaffe RB (2010). Ovarian cancer stem-like side-population cells are tumourigenic and chemoresistant. *British Journal of Cancer*.

[30] Anderson ME (1998). Glutathione: an overview of biosynthesis and modulation. *Chemico-Biological Interactions*.

[31] Kamazawa S, Kigawa J, Kanamori Y (2002). Multidrug resistance gene-1 is a useful predictor of paclitaxel-based chemotherapy for patients with ovarian cancer. *Gynecologic Oncology*.

[32] Backos DS, Franklin CC, Reigan P (2012). The role of glutathione in brain tumor drug resistance. *Biochem Pharmacol*.

[33] Jedlitschky G, Leier I, Buchholz U, Center M, Keppler D (1994). ATP-dependent transport of glutathione S-conjugates by the multidrug resistance-associated protein. *Cancer Research*.

[34] Colvin OM, Friedman HS, Gamcsik MP, Fenselau C, Hilton J (1993). Role of glutathione in cellular resistance to alkylating agents. *Advances in Enzyme Regulation*.

[35] Lessard J, Sauvageau G (2003). Bmi-1 determines the proliferative capacity of normal and leukaemic stem cells. *Nature*.

[36] Liu J, Cao L, Chen J (2009). Bmi1 regulates mitochondrial function and the DNA damage response pathway. *Nature*.

[37] Li J, Gong LY, Song LB (2010). Oncoprotein Bmi-1 renders apoptotic resistance to glioma cells through activation of the IKK-nuclear factor-*κ*B pathway. *American Journal of Pathology*.

[38] Guo BH, Feng Y, Zhang R (2011). Bmi-1 promotes invasion and metastasis, and its elevated expression is correlated with an advanced stage of breast cancer. *Molecular Cancer*.

[39] Wang E, Bhattacharyya S, Szabolcs A (2011). Enhancing chemotherapy response with Bmi-1 silencing in ovarian cancer. *PLoS ONE*.

[40] Wallace-Brodeur RR, Lowe SW (1999). Clinical implications of p53 mutations. *Cellular and Molecular Life Sciences*.

[41] Fraser M, Bai T, Tsang BK (2008). Akt promotes cisplatin resistance in human ovarian cancer cells through inhibition of p53 phosphorylation and nuclear function. *International Journal of Cancer*.

[42] Nikolaev AY, Li M, Puskas N, Qin J, Gu W (2003). Parc: a cytoplasmic anchor for p53. *Cell*.

[43] Woo MG, Xue K, Liu J (2012). Calpain-mediated processing of p53-associated parkin-like cytoplasmic protein (PARC) affects chemosensitivity of human ovarian cancer cells by promoting p53 subcellular trafficking. *The Journal of Biological Chemistry*.

[44] Herr I, Debatin KM (2001). Cellular stress response and apoptosis in cancer therapy. *Blood*.

[45] Gillet JP, Gottesman MM (2010). Mechanisms of multidrug resistance in cancer. *Methods in Molecular Biology*.

[46] Ernst R, Kueppers P, Stindt J, Kuchler K, Schmitt L (2010). Multidrug efflux pumps: substrate selection in ATP-binding cassette multidrug efflux pumps-first come, first served?. *FEBS Journal*.

[47] Juliano RL, Ling V (1976). A surface glycoprotein modulating drug permeability in Chinese hamster ovary cell mutants. *Biochimica et Biophysica Acta*.

[48] Surowiak P, Materna V, Kaplenko I (2006). ABCC2 (MRP2, cMOAT) can be localized in the nuclear membrane of ovarian carcinomas and correlates with resistance to cisplatin and clinical outcome. *Clinical Cancer Research*.

[49] Zhou S, Schuetz JD, Bunting KD (2001). The ABC transporter Bcrp1/ABCG2 is expressed in a wide variety of stem cells and is a molecular determinant of the side-population phenotype. *Nature Medicine*.

[50] Alvi AJ, Clayton H, Joshi C (2003). Functional and molecular characterisation of mammary side population cells. *Breast Cancer Research*.

[51] Hosonuma S, Kobayashi Y, Kojo S (2011). Clinical significance of side population in ovarian cancer cells. *Human Cell*.

[52] van Herwaarden AE, Wagenaar E, Karnekamp B, Merino G, Jonker JW, Schinkel AH (2006). Breast cancer resistance protein (Bcrp1/Abcg2) reduces systemic exposure of the dietary carcinogens aflatoxin B1, IQ and Trp-P-1 but also mediates their secretion into breast milk. *Carcinogenesis*.

[53] Aguilar-Gallardo C, Rutledge EC, Martinez-Arroyo AM (2012). Overcoming challenges of ovarian cancer stem cells: novel therapeutic approaches. *Stem Cell Reviews*.

[54] Wender PA, Galliher WC, Bhat NM (2012). Taxol-oligoarginine conjugates overcome drug resistance in-vitro in human ovarian carcinoma. *Gynecologic Oncology*.

[55] Arai F, Hirao A, Ohmura M (2004). Tie2/angiopoietin-1 signaling regulates hematopoietic stem cell quiescence in the bone marrow niche. *Cell*.

[56] Liu Y, Elf SE, Miyata Y (2009). p53 regulates hematopoietic stem cell quiescence. *Cell Stem Cell*.

[57] Asai T, Liu S Y, Di Giandomenico S (2012). Necdin, a p53 target gene, regulates the quiescence and response to genotoxic stress of hematopoietic stem/progenitor cells. *Blood*.

[58] Hock H, Hamblen MJ, Rooke HM (2004). Gfi-1 restricts proliferation and preserves functional integrity of haematopoietic stem cells. *Nature*.

[59] Du J, Chen Y, Li Q (2012). HIF-1alpha deletion partially rescues defects of hematopoietic stem cell quiescence caused by Cited2 deficiency. *Blood*.

[60] Hua S, Xiaotao X, Renhua G (2012). Reduced miR-31 and let-7 maintain the balance between differentiation and quiescence in lung cancer stem-like side population cells. *Biomedicine & Pharmacotherapy*.

[61] Scott AM, Wolchok JD, Old LJ (2012). Antibody therapy of cancer. *Nature Reviews Cancer*.

[62] Burgos-Ojeda D, Rueda BR, Buckanovich RJ (2012). Ovarian cancer stem cell markers: prognostic and therapeutic implications. *Cancer Letters*.

[63] Ferrandina G, Bonanno G, Pierelli L (2008). Expression of CD133-1 and CD133-2 in ovarian cancer. *International Journal of Gynecological Cancer*.

[64] Long H, Xie R, Xiang T (2012). Autocrine CCL5 signaling promotes invasion and migration of CD133 (+) ovarian cancer stem-like cells via NF-kappaB-mediated MMP-9 upregulation. *Stem Cells*.

[65] Baba T, Convery PA, Matsumura N (2009). Epigenetic regulation of CD133 and tumorigenicity of CD133+ ovarian cancer cells. *Oncogene*.

[66] Smith LM, Nesterova A, Ryan MC (2008). CD133/prominin-1 is a potential therapeutic target for antibody-drug conjugates in hepatocellular and gastric cancers. *British Journal of Cancer*.

[67] Naor D, Sionov RV, Ish-Shalom D (1997). CD44: structure, function, and association with the malignant process. *Advances in Cancer Research*.

[68] Marhaba R, Klingbeil P, Nuebel T, Nazarenko I, Buechler MW, Zoeller M (2008). CD44 and EpCAM: cancer-initiating cell markers. *Current Molecular Medicine*.

[69] Meng E, Long B, Sullivan P (2012). CD44+/CD24- ovarian cancer cells demonstrate cancer stem cell properties and correlate to survival. *Clinical & Experimental Metastasisdoi*.

[70] Heider KH, Kuthan H, Stehle G, Munzert G (2004). CD44v6: a target for antibody-based cancer therapy. *Cancer Immunology, Immunotherapy*.

[71] Ghosh SC, Alpay SN, Klostergaard J (2012). CD44: a validated target for improved delivery of cancer therapeutics. *Expert Opinion on Therapeutic Targets*.

[72] Börjesson PKE, Postema EJ, Roos JC (2003). Phase I therapy study with 186Re-labeled humanized monoclonal antibody BIWA 4 (Bivatuzumab) in patients with head and neck squamous cell carcinoma. *Clinical Cancer Research*.

[73] Casagrande F, Cocco E, Bellone S (2011). Eradication of chemotherapy-resistant CD44+ human ovarian cancer stem cells in mice by intraperitoneal administration of Clostridium perfringens enterotoxin. *Cancer*.

[74] Orian-Rousseau V (2010). CD44, a therapeutic target for metastasising tumours. *European Journal of Cancer*.

[75] Kristiansen G, Sammar M, Altevogt P (2004). Tumour biological aspects of CD24, a mucin-like adhesion molecule. *Journal of Molecular Histology*.

[76] Gao MQ, Choi YP, Kang S, Youn JH, Cho NH (2010). CD24+ cells from hierarchically organized ovarian cancer are enriched in cancer stem cells. *Oncogene*.

[77] Surowiak P, Materna V, Kaplenko I (2006). Unfavorable prognostic value of CD24 expression in sections from primary and relapsed ovarian cancer tissue. *International Journal of Gynecological Cancer*.

[78] Choi YL, Kim SH, Shin YK (2005). Cytoplasmic CD24 expression in advanced ovarian serous borderline tumors. *Gynecologic Oncology*.

[79] Bretz NP, Salnikov AV, Perne C (2012). CD24 controls Src/STAT3 activity in human tumors. *Cellular and Molecular Life Sciences*.

[80] Su D, Deng H, Zhao X (2009). Targeting CD24 for treatment of ovarian cancer by short hairpin RNA. *Cytotherapy*.

[81] Miettinen M, Lasota J (2005). KIT (CD117): a review on expression in normal and neoplastic tissues, and mutations and their clinicopathologic correlation. *Applied Immunohistochemistry and Molecular Morphology*.

[82] Bapat SA, Mali AM, Koppikar CB, Kurrey NK (2005). Stem and progenitor-like cells contribute to the aggressive behavior of human epithelial ovarian cancer. *Cancer Research*.

[83] Luo L, Zeng J, Liang B (2011). Ovarian cancer cells with the CD117 phenotype are highly tumorigenic and are related to chemotherapy outcome. *Experimental and Molecular Pathology*.

[84] Raspollini MR, Amunni G, Villanucci A, Baroni G, Taddei A, Taddei GL (2004). c-KIT expression and correlation with chemotherapy resistance in ovarian carcinoma: an immunocytochemical study. *Annals of Oncology*.

[85] Chau WK, Ip CK, Mak AS (2012). c-Kit mediates chemoresistance and tumor-initiating capacity of ovarian cancer cells through activation of Wnt/beta-catenin-ATP-binding cassette G2 signaling. *Oncogene*.

[86] Schilder RJ, Sill MW, Lee RB (2008). Phase II evaluation of imatinib mesylate in the treatment of recurrent or persistent epithelial ovarian or primary peritoneal carcinoma: a gynecologic oncology group study. *Journal of Clinical Oncology*.

[87] Patel BB, He YA, Li XM (2008). Molecular mechanisms of action of imatinib mesylate in human ovarian cancer: a proteomic analysis. *Cancer Genomics and Proteomics*.

[88] Imrich S, Hachmeister M, Gires O (2012). EpCAM and its potential role in tumor-initiating cells. *Cell Adhesion & Migration*.

[89] Pauli C, Münz M, Kieu C (2003). Tumor-specific glycosylation of the carcinoma-associated epithelial cell adhesion molecule EpCAM in head and neck carcinomas. *Cancer Letters*.

[90] Gosens MJEM, Van Kempen LCL, Van De Velde CJH, Van Krieken JHJM, Nagtegaal ID (2007). Loss of membranous Ep-CAM in budding colorectal carcinoma cells. *Modern Pathology*.

[91] Baeuerle PA, Gires O (2007). EpCAM (CD326) finding its role in cancer. *British Journal of Cancer*.

[92] Thiery JP, Acloque H, Huang RYJ, Nieto MA (2009). Epithelial-mesenchymal transitions in development and disease. *Cell*.

[93] Sebastian M, Kuemmel A, Schmidt M, Schmittel A (2009). Catumaxomab: a bispecific trifunctional antibody. *Drugs of Today*.

[94] Seimetz D, Lindhofer H, Bokemeyer C (2010). Development and approval of the trifunctional antibody catumaxomab (anti-EpCAM×anti-CD3) as a targeted cancer immunotherapy. *Cancer Treatment Reviews*.

[95] Marchitti SA, Brocker C, Stagos D, Vasiliou V (2008). Non-P450 aldehyde oxidizing enzymes: the aldehyde dehydrogenase superfamily. *Expert Opinion on Drug Metabolism and Toxicology*.

[96] Landen CN, Goodman B, Katre AA (2010). Targeting aldehyde dehydrogenase cancer stem cells in ovarian cancer. *Molecular Cancer Therapeutics*.

[97] Saw YT, Yang J, Ng SK (2012). Characterization of aldehyde dehydrogenase isozymes in ovarian cancer tissues and sphere cultures. *BMC Cancer*.

[98] Silva IA, Bai S, McLean K (2011). Aldehyde dehydrogenase in combination with CD133 defines angiogenic ovarian cancer stem cells that portend poor patient survival. *Cancer Research*.

[99] Liu P, Brown S, Goktug T (2010). Cytotoxic effect of disulfiram/copper on human glioblastoma cell lines and ALDH-positive cancer-stem-like cells. *British Journal of Cancer*.

[100] Khanna M, Chen CH, Kimble-Hill A (2011). Discovery of a novel class of covalent inhibitor for aldehyde dehydrogenases. *The Journal of Biological Chemistry*.

[101] Peng S, Maihle NJ, Huang Y (2010). Pluripotency factors Lin28 and Oct4 identify a sub-population of stem cell-like cells in ovarian cancer. *Oncogene*.

[102] Masiakos PT, MacLaughlin DT, Maheswaran S (1999). Human ovarian cancer, cell lines, and primary ascites cells express the human Mullerian inhibiting substance (MIS) type II receptor, bind, and are responsive to MIS. *Clinical Cancer Research*.

[103] Wei X, Dombkowski D, Meirelles K (2010). Müllerian inhibiting substance preferentially inhibits stem/progenitors in human ovarian cancer cell lines compared with chemotherapeutics. *Proceedings of the National Academy of Sciences of the United States of America*.

[104] Sell S (2004). Stem cell origin of cancer and differentiation therapy. *Critical Reviews in Oncology/Hematology*.

[105] Yin G, Alvero AB, Craveiro V (2013). Constitutive proteasomal degradation of TWIST-1 in epithelial-ovarian cancer stem cells impacts differentiation and metastatic potential. *Oncogene*.

[106] Jain AK, Allton K, Iacovino M (2012). p53 regulates cell cycle and microRNAs to promote differentiation of human embryonic stem cells. *PLoS Biology*.

[107] Yu Z, Li Y, Fan H (2012). miRNAs regulate stem cell self-renewal and differentiation. *Frontiers in Genetics*.

[108] Gudas LJ, Wagner JA (2011). Retinoids regulate stem cell differentiation. *Journal of Cellular Physiology*.

[109] Lim YC, Kang HJ, Kim YS (2012). All-trans-retinoic acid inhibits growth of head and neck cancer stem cells by suppression of Wnt/beta-catenin pathway. *European Journal of Cancer*.

[110] Soignet SL, Benedetti F, Fleischauer A (1998). Clinical study of 9-cis retinoic acid (LGD1057) in acute promyelocytic leukemia. *Leukemia*.

[111] Whitworth JM, Londono-Joshi AI, Sellers JC (2012). The impact of novel retinoids in combination with platinum chemotherapy on ovarian cancer stem cells. *Gynecologic Oncology*.

[112] Ruiz-Vela A, Aguilar-Gallardo C, Martinez-Arroyo AM (2011). Specific unsaturated fatty acids enforce the transdifferentiation of human cancer cells toward adipocyte-like cells. *Stem Cell Reviews*.

[113] Ruiz-Vela A, Aguilar-Gallardo C, Simón C (2010). Building a framework for embryonic microenvironments and cancer stem cells. *Stem Cell Reviews and Reports*.

[114] Li HJ, Reinhardt F, Herschman HR (2012). Cancer-stimulated mesenchymal stem cells create a carcinoma stem cell niche via prostaglandin E2 signaling. *Cancer Discovery*.

[115] Lis R, Touboul C, Raynaud CM (2012). Mesenchymal cell interaction with ovarian cancer cells triggers pro-metastatic properties. *PLoS One*.

[116] Katz E, Skorecki K, Tzukerman M (2009). Niche-dependent tumorigenic capacity of malignant ovarian ascites-derived cancer ceil subpopulations. *Clinical Cancer Research*.

[117] Liang D, Ma Y, Liu J (2012). The hypoxic microenvironment upgrades stem-like properties of ovarian cancer cells. *BMC Cancer*.

[118] LaBarge MA (2010). The difficulty of targeting cancer stem cell niches. *Clinical Cancer Research*.

[119] Bartel DP (2009). MicroRNAs: target recognition and regulatory functions. *Cell*.

[120] Lavon I, Zrihan D, Granit A (2010). Gliomas display a microRNA expression profile reminiscent of neural precursor cells. *Neuro-Oncology*.

[121] van Jaarsveld MTM, Helleman J, Berns EMJJ, Wiemer EAC (2010). MicroRNAs in ovarian cancer biology and therapy resistance. *International Journal of Biochemistry and Cell Biology*.

[122] Xu CX, Xu M, Tan L (2012). MicroRNA MiR-214 regulates ovarian cancer cell stemness by targeting p53/Nanog. *The Journal of Biological Chemistry*.

[123] Cheng W, Liu T, Wan X (2012). MicroRNA-199a targets CD44 to suppress the tumorigenicity and multidrug resistance of ovarian cancer-initiating cells. *The FEBS Journal*.

[124] Wu Q, Guo R, Lin M, Zhou B, Wang Y (2011). MicroRNA-200a inhibits CD133/1+ ovarian cancer stem cells migration and invasion by targeting E-cadherin repressor ZEB2. *Gynecologic Oncology*.

[125] Sarkar FH, Li Y, Wang Z, Kong D, Ali S (2010). Implication of microRNAs in drug resistance for designing novel cancer therapy. *Drug Resistance Updates*.

[126] Zhong X, Li N, Liang S, Huang Q, Coukos G, Zhang L (2010). Identification of microRNAs regulating reprogramming factor LIN28 in embryonic stem cells and cancer cells. *Journal of Biological Chemistry*.

[127] Fendler B, Atwal G (2012). Systematic deciphering of cancer genome networks. *Yale Journal of Biology and Medicine*.

[128] Williamson AJ, Whetton AD (2011). The requirement for proteomics to unravel stem cell regulatory mechanisms. *Journal of Cellular Physiology*.

[129] Vathipadiekal V, Saxena D, Mok SC (2012). Identification of a potential ovarian cancer stem cell gene expression profile from advanced stage papillary serous ovarian cancer. *PLoS One*.

